# Domestic

**Published:** 1841-03-06

**Authors:** 


### DOMESTIC.

*Louisville Medical Institute.*—This institution appears to be advancing rapidly in its career of prosperity. Two hundred and five matriculants are announced in the catalogue just issued.

*Professor Drake's Introductory Lecture.*—A very interesting discourse addressed to the class at Louisville, on the importance of clinical medicine and pathology. From it we learn incidentally that, in addition to the facilities previously afforded by the Louisville Institute, an amphitheatre has been erected, devoted to the study of clinical medicine, pathological anatomy, and operative surgery.

*Annual Report of the Board of Trustees of the Massachusetts General Hospital for the year* 1840.—We have received from Dr. Bell a copy of the above report. It appears that the number of patients admitted into the hospital proper during the year 1840, was 362, of whom 168 were free patients. The weekly expenditure for each patient was $4 32; this is extremely high, but of course it must be justified by the large proportion of paying patients, many of them in private rooms.

The report of the insane asylum is the twenty-third in number. The number admitted in the year, or remaining at the beginning of it, was 263. During the same period the discharged as recovered were 75, much improved 12, improved 20, not improved 18, dead 13, remaining 125.

The per centage of recoveries for six years is as follows:

| Year. | Per cent. of recoveries. | Per cent. of deaths of discharged. | Per cent. of deaths of all under care. |
|---|---|---|---|
| 1835 | 53.5 | 13. | 6.1 |
| 1836 | 57.1 | 8.9 | 5.5 |
| 1837 | 68.6 | 7.6 | 4.2 |
| 1838 | 56.4 | 9.1 | 5.4 |
| 1839 | 59. | 8.5 | 4.4 |
| 1840 | 54.3 | 9.4 | 5. |

From these statistics it appears, that during the four years of the direction of its present superintendent, the proportion of recoveries of all cases, old and recent, without allowance for those proving "unfit," or not proper subjects, or those who have died under care, or those who have been prematurely removed before the event was known, has been about sixty in the hundred, and the proportion of deaths between four and five in the hundred.

The remarks of Dr. Bell relative to the statistics of the insane, we fully accord with. We cannot admit the accuracy of many reports on this matter. Not that any attempt is made by the authors of them to deceive the public; but they certainly receive rather hastily the evidence of recovery, and are evidently actuated by a strong desire to make the proportion of successful cases appear as great as possible. Whenever this desire is apparent in a report, an additional amount of testimony must be brought forward to remove the impression that a too favourable view is taken by the author.

Besides, in most of the highly favourable reports, we have not enough of the modifying circumstances, such as the paroxysmal character of many cases, the fact that a large proportion of cases get well only to a certain point, a fact very properly insisted upon by some French writers, &c., all matters important to a correct view of the subject.

The remarks of Dr. Bell are as follows:

"During the last years, I have not attempted to draw the line of demarcation between *recent* and *old* cases, as experience has satisfied me that any attempt to decide upon the duration of time before admission, that the patient has been afflicted, is exceedingly fallacious, giving a mere approximation to the fact. It is liable to the same objection which obtains in essaying to specify the causes of disease, that of giving an apparently mathematical and certain aspect to facts, so involved in doubt, so complicated and vacillating, that they really have nothing like fixedness or certainty.

"The records of this Asylum justify the declaration that *all cases certainly recent*, that is, whose origin does not either directly or obscurely run back more than a year, recover under a fair trial. This is the general law; the occasional instances to the contrary are the exception.

"During some years, as in 1838 for illustration, we have had so entirely the co-operation of friends in a due perseverance that we should have been able to report the recovery of 100 per cent. of cases presumed to be recent. Again, in another year, the premature withdrawal of half a dozen cases before we regarded them as recovered, although their friends might so consider them, would present an entire different view of the curability of insanity in two different years, as far as figures express the fact, although the actual results might be identical. So the general expression of the fact that sixty per cent. of all cases have recovered during the last four years, is of no importance as a means of drawing any conclusions, except as to the different periods of the same institution. In this commonwealth, where the value of hospitals for the insane has been established for over twenty years, and the community is so generally advised of the infinite importance of early subjection to treatment, the results of institutions must be far more favourable than in communities where all this acquaintance and public confidence has yet to be acquired. This is strongly illustrated in contrasting the first and last five years of this Asylum, showing a difference of over a hundred per cent., which is undoubtedly ascribable mostly to this fact of more recent admissions, and some changes in the regulations in regard to hasty removals.

"There is, I am satisfied, no institution in the world, which upon the whole receives its cases with less delay after disease has proved intractable at home, than this. Its *private* character, however, subjects it necessarily to some drawbacks upon its ratio of recoveries. Patients are only retained so long as friends elect;—there is no statutory provision compelling the institution to receive, or preventing the removal of any patient at any time. Hence infirmity of purpose, want of persevering faith, or still more frequently want of pecuniary means, often occasion difference of results in our annual summing up, by the removal of recoverable cases, to an extent which proves the absurdity of the statistics, except as a means of comparison at different epochs of the same establishment.

"Again, in the mortality and restorative results of various institutions, how great a difference should be looked for from difference of regulations as to admissions—in an institution, for example, receiving only cases of violent excitement and high action, and in one where no restriction as to the patient's condition exists, where the exhausted, the aged, the epileptic and the hopeless are brought merely to relieve friends, or in the desperate hope that some new appliances may renovate the exhausted system.

"I have been induced to suggest these hints upon the fallaciousnes of the ordinary statistics published, when regarded irrespective of the varying and modifying circumstances, which, in this country at least, prevent any just comparison from being made in the mere abstract result of figures, not from any apprehension that our Asylum may not appear as well on paper as any other, for the influence of its happy position in a community where the insane, who belong to the classes from which its inmates are derived, as a general rule, are received speedily after a decided outbreak of excitement, always has afforded a larger proportion of curable subjects than I have noticed in any other. But during the past year the attempt has been made (with the best intentions I doubt not) to aggregate the mere figured statistics of the various American hospitals for the insane, and deduce conclusions from them, without reference to their difference of position or of regulations as to receiving, rejecting, or dismissing patients. In such statistics injury must be done to some whose apparent results may not be so honorable as the real, of which only those who are informed of all the particulars of difference, a small portion of the public of course, can discriminate and arrive at an accurate judgment. A single illustration will perhaps place the absurdity of the common expressed statistics of insane hospitals in a stronger light. A new institution in a state where the patients are committed by the order of a judge of some court, is capable of receiving a hundred patients. During its first or second year, or until its accommodations are filled, it might be that its only patients discharged would be the recovered, if its managers or directors should so decide, for in the supposed case the friends have no voice in the decision. Of course its recoveries of all cases, old and recent, would be 100 per cent. And subsequently its per centages would depend exactly upon the number of harmless or incurable or inconvenient subjects which its directors should judge it expedient to dismiss!

"A few years since it became necessary, in order to accommodate the urgent claims of recent cases, for the Trustees to cast their view over the entire class of old cases, many of whom had been in this Asylum from its opening, to ascertain how many and which could be dismissed with least injury to themselves or to the comfort of their friends. So many were dismissed under this resolution "not improved," as to make in that year fifteen or twenty per cent. difference in the apparent results!

"As far as this Asylum is concerned in the apparent bearings of statistical returns, the favourable circumstances in which it stands as before alluded to, prevent any danger of its suffering from comparison of figures;—for in the view of these it is believed that no published returns present more decided favourable results, but feeling unwilling to reap honours not fully deserved, it has been felt due to the sanctity of scientific truth and to justice as regards the new institutions spring up every where around, that the community should be set right on this subject."

The report afterwards alludes to the two classes of establishments for the insane; public hospitals, as is universally the case, or nearly so, in this country, and private asylums, as is the custom in England and many parts of the continent, for all who can pay for them. Dr. Bell decidedly prefers the public institutions; that is, paying institutions, for those who can be supported at the expense of their friends; and for the poor, institutions supported at the public expense. In the main we agree with him, but there is certainly a want of private houses for the insane of a peculiar kind—those of high nervous excitability, a lively imagination, and cultivated taste, with a very limited, though often most distressing mental aberration. Thus individuals often suffer from the circumstances attending public institutions; their number, however, is not great, and a very few private establishments would suffice.

---

Deaths in Boston for the week ending Feb. 20—26. Males 16. Females 10. Causes—consumption 5, intemperance 2, lung fever 4, &c.

### HEALTH OF THE CITY.

INTERMENTS *in the City and Liberties of Philadelphia, from the 20th to the 27th of February*, 1841.

| Diseases. | Adults. | Children. |
|---|---|---|
| Abortion, | 1 | 0 |
| Abscess of lungs, | 0 | 1 |
| Apoplexy, | 2 | 1 |
| Croup, | 0 | 2 |
| Congestion, | 0 | 1 |
| —— of brain, | 1 | 0 |
| Consumption of the lungs, | 18 | 1 |
| Convulsions, | 0 | 6 |
| Constipation, | 1 | 0 |
| Cyanosis, | 0 | 1 |
| Dropsy, | 2 | 0 |
| —— abdominal, | 2 | 1 |
| —— head, | 0 | 6 |
| —— breast, | 1 | 0 |
| Dis'se of the brain, | 0 | 1 |
| Dysentery, | 1 | 0 |
| Debility, | 0 | 4 |
| Erysipelas, | 0 | 1 |
| Fever, scarlet, | 0 | 2 |
| —— remittent, | 1 | 0 |
| —— typhus, | 0 | 1 |
| —— typhoid, | 1 | 0 |
| Inflammation of the brain, | 0 | 4 |
| —— bronchi, | 0 | 4 |
| —— lungs, | 1 | 5 |
| —— stomach and bowels, | 0 | 1 |
| —— bowels, | 0 | 3 |
| | — | — |
| *Carried forward*, | 36 | 46 |
| *Brought forward*, | 36 | 46 |
| Inflammation of the larynx, | 1 | 0 |
| —— peritonæum, | 2 | 0 |
| —— spine, | 0 | 1 |
| Marasmus, | 0 | 3 |
| Old age, | 1 | 0 |
| Scrofula, | 0 | 1 |
| Small pox, | 1 | 2 |
| Still-born, | 0 | 8 |
| Unknown, | 1 | 2 |
| | — | — |
| Total, 105— | 42 | 63 |

---

Of the above, there were under 1 year 36

| | |
|---|---|
| From 1 to 2 | 12 |
| 2 to 5 | 6 |
| 5 to 10 | 4 |
| 10 to 15 | 4 |
| 15 to 20 | 3 |
| 20 to 30 | 8 |
| 30 to 40 | 9 |
| 40 to 50 | 13 |
| 50 to 60 | 1 |
| 60 to 70 | 3 |
| 70 to 80 | 2 |
| 80 to 90 | 3 |
| 90 to 100 | 1 |
| Total, | 105 |

In the above are included 15 people of colour, 9 interments from the alms-house, and 1 from the country.

---

*Weekly Report of Interments in the City and County of New York, from the 20th to the 27th of Feb.*, 1841.—Diseases: Aneurism, 1; asphyxia, 1; burned or scalded, 2; consumption, 32; convulsions, 14; croup or hives, 3; diarrhœa, 2; dropsy, 1; dropsy in the head, 3; dropsy in the chest, 3; dysentery, 1; erysipelas, 1; epilepsy, 1; fever, 4; do. scarlet, 6; do. typhoid, 3; do. puerperal, 2; do. remittent, 4; inflammation, 1; do. of brain, 8; do. of chest, 8; do. of lungs, 15; do. of bowels, 6; do. of stomach, 1; marasmus, 4; measles, 6; old age, 2; organic disease of heart, 1; scirrhus, 1; small pox, 7; teething, 1; unknown, 3; varioloid, 1; worms, 2.

Ages—Of 1 year and under, 30; between 1 and 2, 21; 2 and 5, 22; 5 and 10, 9; 10 and 20, 3; 20 and 30, 17; 30 and 40, 21; 40 and 50, 13; 50 and 60, 7; 60 and 70, 3; 70 and 80, 3; 80 and 90, 2.

38 men—26 women—50 boys—37 girls. Total, 151.

